# Draft genome analysis of a serum-resistant and fourth-generation cephalosporin (cefepime)-resistant *Acinetobacter baumannii* strain TDU5 isolated from Dhaka, Bangladesh

**DOI:** 10.1128/mra.01332-24

**Published:** 2025-03-13

**Authors:** Spencer Mark Mondol, Mohammed Aziz Hossain, Fahim Kabir Monjurul Haque

**Affiliations:** 1 Microbiology Program, Department of Mathematics and Natural Sciences, BRAC University 118864, Dhaka, Bangladesh; 2 Department of Microbiology, University of Dhaka 95324, Dhaka, Bangladesh; 3 Department of Molecular Biology, Umea University 123917, Umeå, Sweden; Department of Biological Sciences, Wellesley College, Wellesley, Massachusetts, USA

**Keywords:** *Acinetobacter*, antibiotic resistance, serum resistance, environmental microbiology, public health, pathogens

## Abstract

We report the draft genome of a cefepime-resistant *A. baumannii* TDU5, isolated from Turag river water in Bangladesh. The strain was serum-resistant and pathogenic. The genome size was 3,586,480 bp with 3,429 coding sequences (CDS). The isolate harbored multiple antimicrobial resistance genes including *bla*_PER-7_, *bla*_ADC-52,_ and *bla*_OXA-91_.

## ANNOUNCEMENT

*Acinetobacter baumannii* (*A. baumannii*) is an opportunistic pathogen associated with severe nosocomial infections, particularly in immunocompromised patients (1, 2). Its ability to evade host immune defenses, including resistance to complement-mediated serum killing, contributes significantly to its pathogenicity. The emergence of resistance to fourth-generation cephalosporins, such as cefepime, further exacerbates the global threat posed by this pathogen, limiting therapeutic options and complicating treatment outcomes (3).

In this study, we have isolated a cefepime-resistant strain of *A. baumannii* from Turag River water near Dhaka Uddan (23.76285 N; 90.33825 E), Bangladesh. A water sample was collected and transported to the lab in sterile 50-mL Falcon conical tubes. The sample was diluted tenfold by mixing 1 mL of the sample with 9 mL of 0.9% sterile saline (NaCl). Afterward, 100 µL of the diluted samples was spread onto Leeds Acinetobacter Agar plate. Isolated single colonies were sub-cultured in nutrient broth. Testing of antimicrobial susceptibility to cefepime was carried out using the disk diffusion procedure following the Kirby Bauer method (4). A serum resistance assay was performed following a described protocol (5). Genomic DNA extraction was carried out using the Genomic DNA Purification Kit for gram-negative bacteria (New England Biolabs, UK), following the manufacturer’s protocol (NEB#T3010). Whole-genome sequencing was conducted on the Illumina platform using the Illumina Miniseq sequencing system at the Bangladesh Council of Scientific and Industrial Research (BCSIR). Paired-end sequencing libraries were prepared using the Nextera XT DNA Library Prep Kit, with an average insert size of 150 bp. The raw FASTQ files were quality-checked using FastQC (v0.11) (6) and trimmed with Trimmomatic (v0.39) (7). The trimming parameters were set to retain reads with a minimum average quality score of 30 and a minimum length of 50 base pairs. High-quality reads were assembled *de novo* using SPAdes (v3.15.4) (8) and annotated with Prokka (9) and RAST (10). The genome was visualized using Proksee (11), and isolate identification was performed with the KmerFinder tool (12). MLST typing was carried out using the MLST 2.0 tool (13). Antibiotic resistance genes (ARGs) were investigated through the CARD (14) server using default parameters. The virulence factor genes (VFGs) and pathogenicity were determined using the VFDB (15) and PathogenFinder (v1.1) (16) tool using default parameters.

**Fig 1:**
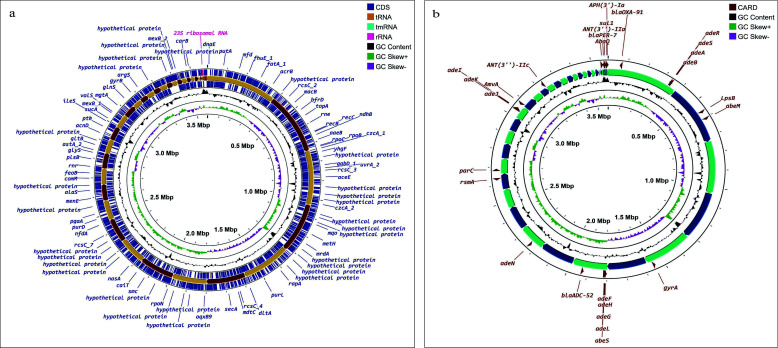
Genomic characterization of *Acinetobacter baumannii* strain TDU5. (a) Circular genome map depicting the organization of coding sequences (CDS), tRNA, rRNA, tmRNA, GC content, and GC skew across the genome. (b) Genome mapping highlighting antimicrobial resistance (AMR) genes identified in strain TDU5. CARD-identified AMR determinants, including resistance gene clusters, are displayed with their respective locations on the genome.

The isolate was found to be resistant to cefepime and exhibited serum resistance as well. A total of 181.1M reads were obtained from Illumina sequencing. The draft genome assembly of TDU5 comprises 3,586,480 base pairs, with detailed features presented in Table 1. The genome map is depicted in Fig. 1a. The cefepime-resistant *A. baumannii* strain TDU5 contains *bla*_PER-7_, *bla*_ADC-52,_ and *bla*_OXA-91_ genes, which are known to be responsible for conferring resistance to different beta-lactam antibiotics including cephalosporins (Fig. 1b). Also, mutations in *gyr*A (S81L) and *par*C (V104I and D105E) genes were also detected. Several virulence factor genes, notably *bas*A, *bas*B, *bau*A, *bau*B, *ent*E, *lps*B, *lpx*A, *lpx*C, *lpx*D, *omp*A, and *plc*D, were detected. Also, the csu operon (csuABCDE) and pga operon (pgaABCD) were found. Along with that, the *pbp*G gene was detected to have a predicted role in serum resistance. The strain was detected to have 86.3% probability of being a human pathogen having 624 matched pathogenic families. This study shed light on uncovering the genomic characteristics of a pathogenic *A. baumannii* strain isolated from an environmental source, which underscored the importance of the implementation of the “One Health” approach in Bangladesh.

**TABLE 1:** General features of the draft genome of *Acinetobacter baumannii* TDU5

General features	*Acinetobacter baumannii* TDU5
Completeness of genome	100
Coarse consistency	99.3
Fine consistency	98.5
Contigs	53
GC Content	38.91
Genome length (bp)	3,586,480
CDS	3,429
CDS ratio	0.9560906
Hypothetical CDS	748
N50 value	213009
L50 value	6
RNA	Three rRNA, 35 tRNA, and one tmRNA
MLST	234
Accession	JBJYSV000000000

## Data Availability

The annotated draft genome sequence has been deposited in the NCBI under the DDBJ/ENA/GenBank accession number JBJYSV000000000. The raw data are available in the NCBI Sequence Read Archive under accession number SRR31940119.
